# Dopamine and serotonin in fear extinction: some key questions to be addressed

**DOI:** 10.3934/Neuroscience.2020014

**Published:** 2020-07-22

**Authors:** Carmelo M Vicario, Gabriella Martino

**Affiliations:** 1 Department of Cognitive Sciences, Psychology, Education and Cultural Studies, University of Messina, Messina, Italy; 2 Department of Clinical and Experimental Medicine, University of Messina, Messina, Italy

**Keywords:** dopamine, serotonin, fear extinction, learning, consolidation

*Fear extinction* learning is considered a key process for a successful treatment of anxiety disorders and the post-traumatic stress disorder (PTSD) [1], two mental health disorders that might affect several clinical conditions including diabetes [2]–[6], which involves the gradual reduction of the fear response when the conditioned stimulus—a neutral stimulus associated with an aversive stimulus (i.e. the unconditioned stimulus)—is repeatedly presented without the aversive outcome.

A recent review [7] highlighted the role of the dopaminergic system in fear extinction. The involvement of dopamine, a neurobiological mediator classically associated with the experience of reward [8]–[10], in fear extinction [11],[12] is interpreted in line with the suggestion that the omission of an unconditioned adverse stimulus in association with a conditioned (i.e., neutral) stimulus is experienced/interpreted as appetitive event. This interpretation fits with the evidence that the experience of pain relief is associated with a release of dopamine at the ventral tegmental area (VTA) [13], similarly to what happens in response to a reward [14]. Nevertheless, it seems simplistic to talk about the role of dopamine in fear extinction without mentioning the contribution of serotonin, which is crucial in encoding aversive motivational outcomes, and represents the natural opponent of dopaminergic system in conditioning tasks [15]. First of all, evidence exists about an involvement of serotonin in extinction learning [e.g., [16] for a review].

The scientific literature has also shown that in the context of reward and aversion processing both chemical messengers work in antagonistic way [15],[17]. This is suggested by research on Prediction Error (PE): an increase in the activity of dopaminergic system and a decrease in the activity of serotonergic system encode PE associated with undelivered punishment; furthermore, the dopaminergic system is activated when the reward is signaled, while the serotonergic system is activated when the expected reward fails to arrive [15]. This implies that an exhaustive analysis of the role of dopaminergic system in fear extinction should take into account the symmetric involvement of serotonergic system and that, because of this interdependence, it can be technically challenging to clarify whether a fear extinction learning response pattern, generated in an experimental and/or clinical setting, truly reflects the activity of the dopaminergic system, the activity of the serotonergic system, or it is the result of a synergistic interaction between these two systems.

Worthy of mention is also the necessity to clarify whether the role played by dopaminergic and serotoninergic systems in fear extinction learning is inexorably interdependent, or it is possible to identify a degree of autonomy in each system with regard to the process of fear extinction learning. A closer look at the process of fear extinction could add some timely insights in this regard.

When speaking about fear extinction it is important to distinguish between learning and consolidation [18]. Learning represents the first stage of memorization that takes place during wakefulness and is based on the process of encoding. The consolidation represents the next step, a fundamental process for the stabilization of new traces of memory that occurs during sleep. In line with this distinction, the current literature suggests that high activity of serotonergic system could be selectively involved in the consolidation of fear extinction material. This is corroborated by the evidence [19], in animal models (i.e., serotonin transporter knock-out mouse) that the genetically driven loss of serotonin function is associated with a selective deficit in extinction recall (i.e., 24 h later), which reflects the consolidation process. Importantly, no effect was reported in the encoding stage by the authors of this study [19]. This result is confirmed in a further investigation [20] where the stimulation or blockade of serotonin 2A receptor in mice respectively facilitates and impairs the consolidation of fear extinction memory traces with no effects on the process of encoding (see also the study of Barratta et al. [21] showing that high levels of serotonin can be essential for the consolidation phase of fear memories). In contrast with this evidence, the study by Burghardt et al. [22] has shown that chronic administration of citalopram (a serotonin reuptake inhibitor) to rats, given between fear conditioning and extinction (i.e., during the learning stage), impaired acquisition of extinction. In summary, the research discussed above suggests that high levels of serotonin can both facilitate and interfere with fear extinction, depending on which stage of memory is considered. Current evidence on dopaminergic system suggests the potential involvement of this neurotransmitter both in the encoding and the consolidation stages of the fear extinction learning process, as also reported by Kalish et al. [7] in their article [23]. Given these premises, one might speculate the existence of an interaction between dopamine and serotonin during the learning stage, as low levels of the latter monoamine might be necessary for successful fear extinction learning (this can be inferred by the evidence [22] that high levels of serotonin during the learning stage interfere with fear extinction).

In conclusion, the current research leaves open the hypothesis that the effect of dopaminergic system on learning of fear extinction memories could reflects, at least in part, some interaction with serotonergic system. Therefore, both monoamine should be simultaneously considered when speaking about fear extinction. This is supported also at the neural level, as the serotonergic system of the dorsal raphe nucleus [24], which is considered the system that opposes dopaminergic activity [25], has important innervations with several regions of the brain considered crucial in fear extinction learning such as the VTA [26], the Amygdala [27], and the Ventromedial Prefrontal Cortex [27].
